# Supplementary material for: Epigenetic Modifier SETD8 as a Therapeutic Target for High-Grade Serous Ovarian Cancer
Source: Biomolecules. 2020 Dec 16;10(12):1686. doi: 10.3390/biom10121686 (PMC7766894; doi:10.3390/biom10121686)
Supplement: Supplementary file 1 [file biomolecules-10-01686-s001.pdf]

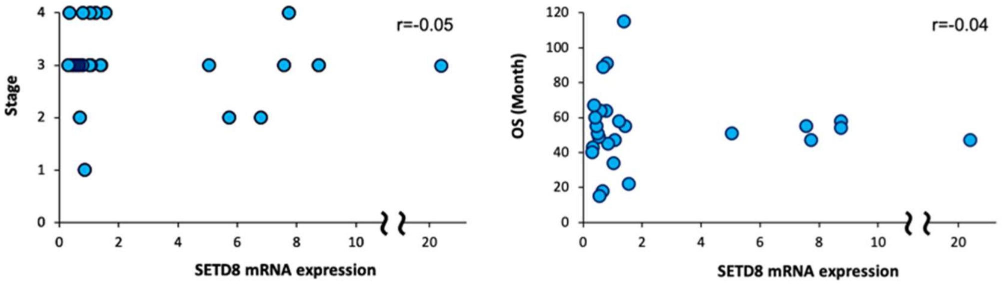

Figure S1. Correlation between the mRNA expression level of SETD8 and the stage/OS of HGSOC patients. There was no correlation between the mRNA expression level of SETD8 and the stage/survival/ of HGSOC patients(n = 34) from Pearson correlation.

Table S1. mRNA expression level of SETD8, stage, and overall survival of HGSOC patients.

No.	1	2	3	4	5	6	7	8	9	10	11	12	13	14	15	16	17	18	19	20	21	22	23	24	25	26	27	28	29	30	31	32	33	34
SETD8 mRNA expression	20.63	8.73	8.73	7.73	7.57	6.79	5.72	5.05	1.55	1.41	1.39	1.21	1.06	1.04	1.03	1.02	0.84	0.80	0.77	0.68	0.66	0.66	0.61	0.60	0.55	0.53	0.49	0.45	0.44	0.42	0.37	0.34	0.33	0.31
Stage	3	3	3	4	3	2	2	3	4	3	3	4	3	3	4	3	1	4	3	2	3	3	3	3	3	3	3	3	3	3	3	3	4	3
OS (Month)	47	58	54	47	55	NA	NA	51	22	55	115	58	47	NA	34	NA	45	91	64	89	18	NA	NA	64	15	49	51	55	NA	60	67	43	NA	40

NA: Not available

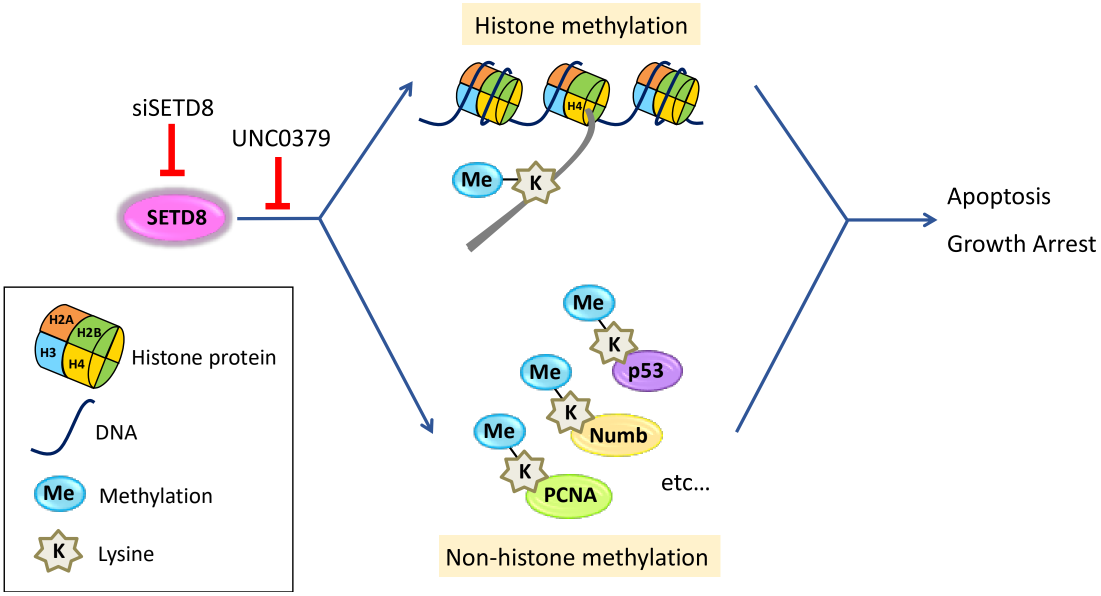

Figure S2. Schematic representation of the role of SETD8 and potential mechanism of pro-apoptotic response induction in HGSOC.
